# Rates and predictors of mental stress in Rwanda: investigating the impact of gender, persecution, readiness to reconcile and religiosity via a structural equation model

**DOI:** 10.1186/1752-4458-8-37

**Published:** 2014-09-03

**Authors:** Lale Heim, Susanne Schaal

**Affiliations:** Department of Psychology, University of Konstanz, 78457 Konstanz, Germany; Department of Psychology, University of Ulm, 89069 Ulm, Germany

**Keywords:** Rwanda, Genocide, Mental stress, Reconciliation, Religiosity

## Abstract

**Background:**

As a consequence of the 1994 Rwandan genocide, prevalences of mental disorders are elevated in Rwanda. More knowledge about determinants of mental stress can help to improve mental health services and treatment in the east-central African country. The present study aimed to investigate actual rates of mental stress (posttraumatic stress disorder, syndromal depression and syndromal anxiety) in Rwanda and to examine if gender, persecution during the genocide, readiness to reconcile as well as importance given to religiosity and quality of religiosity are predictors of mental stress.

**Methods:**

The study comprised a community sample of *N* = 200 Rwandans from Rwanda’s capital Kigali, who experienced the Rwandan genocide. By conducting structured interviews, ten local Master level psychologists examined types of potentially lifetime traumatic events, symptoms of posttraumatic stress disorder (PTSD), depression and anxiety, readiness to reconcile and religiosity. Applying non-recursive structural equation modeling (SEM), the associations between gender, persecution, readiness to reconcile, religiosity and mental stress were investigated.

**Results:**

Respondents had experienced an average number of 11.38 types of potentially lifetime traumatic events. Of the total sample, 11% met diagnostic criteria for PTSD, 19% presented with syndromal depression and 23% with syndromal anxiety. Female sex, persecution and readiness to reconcile were significant predictors of mental stress. Twofold association was found between centrality of religion (which captures the importance given to religiosity) and mental stress, showing, that higher mental stress provokes a higher centrality and that higher centrality reduces mental stress. The variables positive and negative religious functioning (which determine the quality of religiosity) respectively had an indirect negative and positive effect on mental stress.

**Conclusions:**

Study results provide evidence that rates of mental stress are still elevated in Rwanda and that female sex, persecution, readiness to reconcile, centrality and religious functioning are predictors of mental stress. Seventeen years after the genocide, there remains a large gap between the need for and provision of mental health services in Rwanda. Results underline the importance of improving the respective infrastructure, with a focus on the requirements of women and persons, who were persecuted during the genocide. They further highlight that the consideration of readiness to reconcile, centrality and religious functioning in therapeutic interventions can aid mental health in Rwanda.

## Background

In the 1994 Rwandan genocide in one hundred days about 800,000 Tutsis and moderate Hutus were killed and the emotional integrity of millions of Rwandans was destroyed. The perpetrators were not only paid soldiers, but to a great extent ordinary citizens. About 25% of the complete Hutu population, including women and children, joined in the killing [1].

As a consequence of the genocide, rates of mental disorders are elevated in Rwanda. In community samples rates for PTSD range from 24.8% [2] to 46.4% [3], for depression from 15.5% [4] to 46.4% [3] and add up to 58.9% [3] for anxiety symptoms. Across all mentioned studies, women were more affected by mental disorders than men. Pham et al. [2] revealed that persons who identified themselves as Tutsis (the ethnic group that was mainly persecuted during the genocide) had a higher probability of fulfilling symptom criteria for PTSD compared to those who identified themselves as Hutus.

Seventeen years following the genocide, the first objective of this study was to investigate rates of PTSD, syndromal depression and syndromal anxiety in a Rwandan community sample. Our second goal was to investigate correlates and thus potential predictors of mental stress (operationalized as anxiety, depression and PTSD severity) in Rwanda. Based on the results of the above-mentioned studies we expected female sex and persecution during the genocide to be direct predictors of mental stress. As Rwanda is marked by post-conflict conditions and a high religiosity, we further supposed readiness to reconcile and religiosity to be predictive for mental stress.

As a consequence of the severe interpersonal violations during the genocide, reconciliation remains a great challenge in Rwanda. Defined as an interpersonal and reciprocal process [5, 6] that guides the formerly hostile partners to mutual acceptance [7], reconciliation can be impeded by the traumatisation of one of the parties. Research in Rwanda has demonstrated that severity of PTSD [2, 3, 8], depression [3] and anxiety as part of poor mental health [9] is negatively correlated with reconciliation. We thus expected a higher readiness to reconcile to be a predictor for lower mental stress.

In stressful periods of life people tend to refer to religion [10], which offers explanation for human suffering and meaning for loss [11]. Despite the involvement of the Church in the genocide [12], 94% Rwandans are belonging to a Christian church [13] and religion plays an essential role in daily life. To investigate the relation between religiosity and mental stress in the present study, we differentiated importance given to religiosity and quality of religiosity. While the “importance given to religiosity” refers to the extent of religious practice (e.g. church attendance), religious ideology, experience and interest, the “quality of religiosity” refers to the emotional content of religious belief (e.g. positive or negative emotions versus God). With reference to Huber [14] the importance given to religiosity was labeled *centrality*, reflecting the centrality of an individual’s religious construct system in daily life. Regarding the quality of religiosity, we differentiated *positive and negative religious functioning*. While positive religious functioning contained a positive religious coping style and positive emotions towards God, negative religious functioning contained a negative religious coping style and negative emotions towards God (see Methods).

Research in Rwanda provided evidence, that the importance given to religious/spiritual belief is protective for the development of prolonged grief reactions in genocide survivors [15]. It was further found, that members of healing groups with a religious focus present with fewer trauma symptoms than those of secular healing groups [7]. Regarding the relation between centrality and mental stress we thus expected twofold association. With reference to Pargament [10] and Boehnlein [11] it was expected on the one hand, that higher mental stress provokes a higher centrality of religion. On the other hand it was supposed, that higher centrality is protective for mental stress.

Concerning the impact of religious functioning on mental stress a meta-analytic review [16] revealed, that positive religious coping is associated with better and negative religious coping with less psychological adjustment. In line with this result, negative emotions towards God were found to be correlated with lower mental health [17]. Accordingly, we expected positive and negative religious functioning to predict lower and higher mental stress, respectively. Additionally, religious functioning was supposed to exert an indirect influence on mental stress. The association between religious coping and psychological adjustment was found to be stronger in more religious persons [18]. Further, Lawler-Row [19] reported that the relationship between religious coping and depression was partly mediated by self-forgiveness and other-forgiveness. The concept of reconciliation is closely linked to forgiveness [20], which is an integral part of Christianity. Thus, it was hypothesized that the relationship between religious functioning and mental stress was partly mediated by centrality and by readiness to reconcile. Positive religious functioning was expected to have a positive, and negative religious functioning to have a negative impact on centrality as well as on the readiness to reconcile.

## Methods

### Study design and procedure

The study was conducted in January and February of 2011 in Kigali, Rwanda. It was approved by the University of Konstanz Ethical Review Board and the Rwandan Ministry of Science and Technology. All instruments, which had not yet been translated as part of previous studies [3, 15, 21], were translated into Kinyarwanda and back by two independent Rwandans.

A total of 200 persons were interviewed in this study. Eligible participants were residents of five randomly selected quarters of Rwanda’s capital Kigali (Muhima, Ndera, Nyamirambo, Kimisigara and Remera). Interviewees were at least 30 years old at the time of the interview and had stayed in Rwanda during the 1994 genocide. Data were collected using a house-to-house survey. Interviewers started at a convenient point in the respective quarter and approached each subsequent house until the required number of interviews was achieved. Houses were re-approached at a later time if nobody was encountered or available upon the first try. Interviews were carried out individually in a private room in the house. If more than one of the dwellers fulfilled inclusion criteria, one participant was randomly chosen. Prior to the interview, all subjects were fully informed about the study’s aim and procedure as well as about voluntary participation. Participants signed a respective written informed consent and were rewarded with 1,000 Rwandan Francs (about 1.20 Euro).

A team of ten local Master level psychologists carried out the interviews. Each of them conducted 20 interviews and was regularly supervised during the period of data collection. Before data collection, the interviewers had been introduced to the aims and the procedure of this study in an intensive one-day training session. All interviewers had already received extensive training in conducting structured diagnostic interviews and had already conducted interviews as part of previous studies in Rwanda. The interviews lasted about two hours. Six subjects who were approached refused to participate in the trial.

### Measures

Socio-demographic data collected included age, sex, marital status, education, employment and religious affiliation. As it is prohibited to directly name ethnic belongings in Rwanda, we additionally asked each respondent if he or she belonged to the group of persons that had been persecuted during the genocide (Tutsis and moderate Hutus) or to the group that had not been persecuted (Hutus).

The modified Event-Scale [3, 21] was used to assess exposure to types of potentially lifetime traumatic events. This instrument includes 26 items and has a dichotomous response format. PTSD diagnostic status and symptom severity were assessed with the PTSD Symptom Scale-Interview (PSS-I [22]; possible scores range from 0–51; *n* = 199, range: 0–38, *M* = 5.66, *SD* = 7.79, Cronbach’s α = .93), which contains a four-stage response format ranging from 0 *(not at all/only once)* to 3 *(five or more times per week/almost always)*.

Symptoms of depression and anxiety were determined using the Hopkins Symptom Checklist-25 (HSCL-25) [23]. Participants rated 15 depression items and ten anxiety items on a four-point scale ranging from 1 *(not at all bothered)* to 4 *(extremely bothered)* (depression: possible scores range from 15–60; *n* = 197, range: 15–46, *M* = 21.32, *SD* = 7.66, Cronbach’s α = .90; anxiety: possible scores range from 10–40; *n* = 198, range: 10–40, *M* = 15.07, *SD* = 6.44, Cronbach’s α = .92). With reference to Mollica and colleagues [24], a mean score of 1.75 was defined as the cut-off point for syndromal depression and anxiety.

The 15-item Readiness to Reconcile Inventory (RRI [8]; possible scores range from 0–60; *N* = 200, range: 3-52, *M* = 32.40, *SD* = 10.28, Cronbach’s α = .81) was used to measure reconciliation. Items of the RRI are answered on a five-point scale ranging from 0 (*totally disagree*) to 4 (*totally agree*) and capture reconciliation on the macro (political; e.g. “In this country, all humans are treated equally.”), meso (group; e.g. “In Rwandan marriages, group membership plays an important role.”) and micro (individual; e.g. “I am careful towards Rwandans who I don’t know.”) levels.

To measure centrality, we applied the ten-item version of the Centrality of Religiosity Scale (CRS) [14]. The CRS (possible scores range from 10–50; *N* = 200, range: 13–50, *M* = 40.87, *SD* = 7.88, Cronbach’s α = .90) captures centrality on five dimensions (ideology, experience, private practice, public practice and intellect) and items are answered on a five-point scale with a range from 1 to 5 (higher scores indicate higher centrality). Depending on their total sum score, respondents can be divided into non-religious (sum score ≤ 20), religious (21 ≤ sum score ≤ 39) or highly religious (sum score ≥ 40) [14, 25].

The Brief Religious Coping Scale (Brief RCOPE) [26] was used to capture different ways of religious coping. For each of the 14 items respondents rate the extent to which they apply the respective way of religious coping on a four-point scale, ranging from 0 *(not at all)* to 3 *(to a great deal)*. The positive religious coping score (possible scores range from 0–21; *n* = 196, range: 0–21, *M* = 15.84, *SD* = 4.59, Cronbach’s α = .85) is the sum of seven items that assess positive means of religious coping (e.g. “Sought help from God in letting go of my anger.”) and the negative religious coping score (possible scores range from 0–21; *n* = 199, range: 0–21, *M* = 8.13, *SD* = 5.74, Cronbach’s α = .82) is the sum of seven items that assess negative means of religious coping (e.g. “Wondered whether God had abandoned me.”).

Emotions towards God were measured with ten items of the Inventory of Emotions towards God (EtG) [27]. Five items captured positive emotions towards God (reverence, protection, gratitude, hope, release from guilt) and five items captured negative emotions towards God (rage, guilt, failure, shame, anxiety). Respondents were asked the following question: “How often do you experience situations in which you feel the following emotions towards God?” and rated the items on a five-point scale ranging from 1 *(never)* to 5 *(very often)*. By adding up the positive and negative items, a positive emotions towards God score and a negative emotions towards God score were respectively obtained (possible sum scores of the subscales range from 5–25; positive emotions towards God score: *N* = 200, range: 5–25, *M* = 21.54, *SD* = 3.61, Cronbach’s α = .88; negative emotions towards God score: *N* = 200, range: 5–25, *M* = 12.49, *SD* = 5.27, Cronbach’s α = .81).

To control for socially desirable response behavior, the dichotomous lie scale “openness” from the revised Freiburger Personality Inventory was applied (FPI-R [28]; possible scores range from 0-12; *n* = 192; range: 0–12, *M* = 6.85, *SD* = 2.85, reliability (Kuder-Richardson formula) = .76). All diagnostic instruments were administered as clinical interviews.

### Data analysis

Data were analysed with version 20 of the SPSS and AMOS software. The descriptive data are expressed as frequencies (%), mean scores and standard deviations. Chi-square test and t-tests were used to investigate group differences. All reported statistical tests were two-tailed.

**Figure 1:**
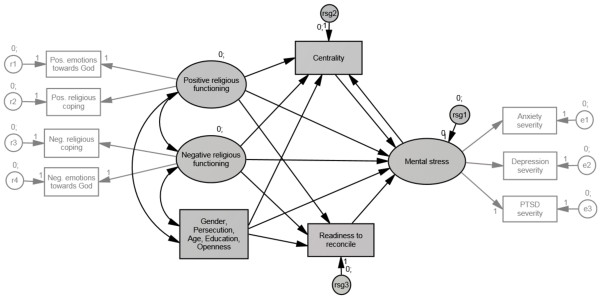
**Initial structural equation model.** Gender, persecution and control variables are summarized in one square.

**Figure 2:**
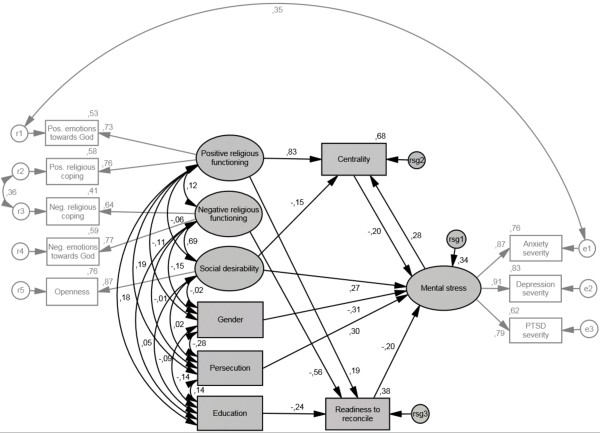
**Final structural equation model with standardized path coefficients.**

We applied non-recursive structural equation modeling with maximum likelihood (ML) to analyse the relationships between the exogenous variables (gender, persecution, positive religious functioning and negative religious functioning plus the control variables age, education, socially desirable response behavior, gender and persecution) and the endogenous variables of readiness to reconcile, centrality of religion and mental stress. The measurement models of positive and negative religious functioning included positive and negative religious coping as well as positive and negative emotions towards God. Anxiety, depression and PTSD severity constituted the reflective indicator variables for mental stress. The lie scale openness functioned as a reflective single indicator for the latent control variable of social desirability. In order to obtain an economic model, we trimmed the initial structural equation model (see Figure 1) by deleting paths with non-significant regression weights (*p* > .10) between exogenous and endogenous variables in a stepwise manner. Coefficients and significances of indirect effects of the final structural equation model (see Figure 2) were examined by applying bootstrapping. For bootstrapping we imputed the data set via regression imputation.

## Results

### Demographic characteristics

The sample consisted of 112 women (56%) and 88 men (44%) with an average age of 43.50 years (*SD* = 10.67, range: 35–94 years). Of the total sample, 113 (56%) respondents indicated that they had been persecuted and 87 (44%) indicated that they had not been persecuted during the genocide. Between these groups, a significant gender difference was found (persecuted females: 68% (*n* = 77), persecuted males: 32% (*n* = 36) vs. non-persecuted females: 40% (*n* = 35), non-persecuted males: 60% (*n* = 52), χ^2^(1, *N* = 200) = 15.54, *p* < .001). With regards to education, 48% (*n* = 96) had no school degree. For the others, the highest level of education completed was primary school for 42% (*n* = 83), secondary school for 5% (*n* = 10), apprenticeship for 3% (*n* = 7) and university for 2% (*n* = 4). The average years of school attended was 5.12 years (*SD* = 4.11, range: 0–18 years). Participants were Catholic (46%, *n* = 91), Protestant (24%, *n* = 49), Adventist (12%, *n* = 24), Muslim (9%, *n* = 18), belonged to another faith community (8%, *n* = 15) or indicated that they did not practice any religion (1%, *n* = 3). The analysis of the centrality scale revealed that 63% (*n* = 127) of the total sample was highly religious, 34% (*n* = 68) was religious and 3% (*n* = 5) was non-religious.

### Trauma exposure and rates of mental stress

Participants were exposed to an average of 11.38 (*SD* = 4.53) types of potentially lifetime traumatic events (*n* = 197, range: 1-22). With a mean number of 12.13 (*SD* = 4.55), females experienced significantly more types of potentially lifetime traumatic events than males (*M* = 10.44, *SD* = 4.33; *t*(195) = 2.64, *p* = .009). The mean number of events experienced was 13.17 (*SD* = 3.99) in the persecuted-group and 9.07 (*SD* = 4.13) in the non-persecuted-group (*t*(195) = −7.05, *p* < .001). Of the total sample, 11% (*n* = 22) met diagnostic criteria for PTSD, 19% (*n* = 37) met clinically significant rates of depression and 23% (*n* = 46) presented with syndromal anxiety. Diagnostic criteria for all three conditions (PTSD, syndromal depression and anxiety) were met by 8% (*n* = 16).

### Structural equation model

Correlations of all manifest variables in the initial structural equation model are presented in Table 1. The final structural equation model fits the data (χ^2^ = 41.34, *df* = 43, *p* = .544; *CMIN/DF* = .96; *RMSEA* = .00; *PCLOSE* = .97) and has a stability index of .06 for the endogenous variables of centrality and mental stress. Coefficients of the final structural equation model can be drawn from Table 2. The proportions of variance explained were *R*^*2*^ = .34 for mental stress, *R*^*2*^ = .68 for centrality and *R*^*2*^ = .38 for readiness to reconcile. With regards to our hypotheses, female sex, persecution and readiness to reconcile were significant predictors of mental stress. Furthermore, we found mental stress to have a significant positive effect on centrality and centrality to have a significant negative effect on mental stress. Positive religious functioning had a direct positive effect on centrality and on readiness to reconcile as well as an indirect negative effect on mental stress (β = -.19, *p* = .003). Negative religious functioning had a direct negative effect on readiness to reconcile and an indirect positive effect on mental stress (β = 11, *p* = .002).

**Table 1:** **Correlations of the manifest variables in the initial structural equation model**

Variable	2	3	4	5	6	7	8	9	10	11	12	13	14
1. Pos. EtG^a^	.57**	.02	-.08	-.05	.09	.01	-.09	-.08	.58**	.18*	-.13	.08	.01
2. Pos. rel. coping^b^	--	.16*	.23**	-.08	.17*	.04	.01	.04	.61**	.01	.07	.14	-.04
3. Neg. EtG^a^		--	.52**	-.10	.02	.17*	.24**	.16*	.11	-.41**	.46**	.03	-.18*
4. Neg. rel. coping^b^			--	-.13	.01	.15*	.20**	.18*	-.03	-.38**	.42**	.05	-.23**
5. Gender^c^				--	-.28**	-.34**	-.36**	-.32**	-.20**	.11	-.01	-.10	.03
6. Persecution^d^					--	.22**	.27**	.26**	.26**	.05	.03	.13	-.02
7. Anxiety severity						--	.80**	.68**	.06	-.23**	.27**	.05	-.13
8. Depression severity							--	.71**	.08	-.29**	.27**	.03	-.08
9. PTSD severity								--	.11	-.29**	.28**	.06	-.13
10. Centrality									--	.01	-.07	.21**	.00
11. Readiness to reconcile										--	-.34**	-.23**	.12
12. Openness											--	-.07	-.32**
13. Education												--	-.20**
14. Age													--

*Note. N* = 200.

^a^Pos. EtG/Neg. EtG = Positive emotions towards God/Negative emotions towards God.

^b^Pos. rel. coping/Neg. rel. coping = Positive religious coping/Negative religious coping.

^c^Gender coded as female = 0, male = 1. ^d^Persecution coded as not persecuted = 0, persecuted = 1.

*p < .05. **p < .01.

**Table 2:** **Coefficients of the final structural equation model**

Dependent variable	Independent variable	***B***	***SE B***	β
Mental stress	Gender^a^	−3.80***	.85	-.31
Mental stress	Persecution^b^	3.67***	.86	.30
Mental stress	Social desirability	.66**	.20	.27
Mental stress	Readiness to reconcile	-.12**	.04	-.20
Mental stress	Centrality	-.16*	.07	-.20
Centrality	Mental stress	.35***	.10	.28
Centrality	Positive RF^c^	2.46***	.26	.83
Centrality	Social desirability	-.48*	.22	-.15
Readiness to reconcile	Negative RF^c^	−1.43***	.22	-.56
Readiness to reconcile	Positive RF^c^	.75**	.28	.19
Readiness to reconcile	Education	-.60***	.16	-.24

*Note. N* = 200*. R* ^*2*^_*Mental stress*_ = .34, *R* ^*2*^_*Centrality*_ = .68, *R* ^*2*^_*Readiness to reconcile*_ = .38.

^a^Gender coded as female = 0, male = 1.

^b^Persecution coded as not persecuted = 0, persecuted = 1. ^c^RF = religious functioning.

*p < .05. **p < .01. ***p < .001.

## Discussion

The first objective of the present study was to investigate rates of mental stress in Rwanda. Results provided evidence, that 17 years after the genocide rates of PTSD, syndromal depression and syndromal anxiety are still elevated in Rwanda: of the total sample 11% met diagnostic criteria for PTSD, 19% presented with syndromal depression and 23% with syndromal anxiety. The second goal was to investigate predictors for mental stress. Female sex, persecution during the genocide and readiness to reconcile were found to be predictive for mental stress. Data further revealed a twofold relationship between centrality and mental stress. Positive and negative religious functioning respectively had an indirect negative and positive effect on mental stress.

Despite the high prevalence of mental disorders, access to qualified mental health services and treatment is limited in Rwanda. In 2011, the year in which the present study was conducted, there were only five psychiatrists and one neuropsychiatric hospital based in Rwanda [29]. Given their high affection of mental disorders, women and persons, who were persecuted during the genocide, form particular risk groups in Rwanda. To facilitate the psychiatric supply of women, who became victims of collective sexual violence during the genocide it is proposed to improve access to qualified mental health services and to ameliorate the equipment of rural health centres with qualified staff [30]. Respective measures would also benefit the larger groups of women and persons persecuted during the genocide, who suffer from mental stress.

According to our results readiness to reconcile is another factor, which contributes to less mental stress. Retributive and restorative justice, reparations, sites and practices of remembrance as well as educational and therapeutic measures are approaches to promote reconciliation [5]. Most of these interventions refer to the macro level of reconciliation and might thus contribute to a general reduction of mental stress in Rwanda. With regards to the individual and group level, educational and therapeutic measures may help to reduce mental stress. Interventions, which include genocide- and trauma education as well as perspective taking exist [31] and can serve as examples for respective reconciliation-sensitive approaches.

Data also confirmed the twofold association between centrality and mental stress, indicating, that higher mental stress provokes a higher importance given to religion and that a higher importance given to religion results in less mental stress. Pargament [10] argues, that religion is an alternative resource that offers orientation and stability in face of disturbing experiences for those who have less access to secular resources and power. Contrarily to the deficient psychiatric infrastructure*,* churches, missionaries and church-linked nongovernmental organizations are numerous in developing Rwanda [32]. Especially smaller Christian fellowships are nearby and low-threshold in offering therapeutic spiritual support [33]. Thus, the finding that mental stress leads to higher centrality might be explained by the high availability of churches and church-related offers in Rwanda. The sense of control and meaning, offered to believers by religion [34] and religious social support, which was found to be positively associated with recovery from mental illness [35], may explain the negative impact of centrality on mental stress. However, church practices are not only positive in Rwanda. Though very poor, believers are frequently asked to pay high financial contributions. Additionally, church-staff is not usually trained in mental health care and can fail to respond adequately to the therapeutic needs of their followers. Given the importance of religion and thus of churches in Rwanda, legal control of church practices and training of church staff in mental health issues may contribute to improve mental health care in Rwanda.

Regarding the quality of religious belief, neither positive nor negative religious functioning exerted direct influence on mental stress. Yet, mediated by centrality and readiness to reconcile, positive religious functioning indirectly predicted lower mental stress and, mediated by readiness to reconcile, negative religious functioning indirectly predicted higher mental stress. Attachment theory and notion of God can help to explain these findings. Positive religious functioning contains positive religious coping; thus, a secure relationship with God and a feeling of spiritual bond with others, including readiness for religious forgiveness [26]. It is also based on positive emotions towards God – among others, the release of guilt [27]. Contrarily, negative religious functioning is marked by an insecure relationship with God and a precarious worldview, including punitive religious reappraisals [26]. It further contains negative emotions towards God including feelings of guilt [27]. It is thus apparent that positive religious functioning stimulates, while negative religious functioning decelerates readiness to reconcile. It is thereby also comprehensible that positive religious functioning reinforces centrality, as the latter is experienced as comfortable, while negative religious functioning does not strengthen, nor – given the belief in a strict and punishing God, who might not accept reduced religious praxis – weaken centrality. Thus, by strengthening centrality and readiness to reconcile, positive religious functioning reduces mental stress, while negative religious functioning fosters mental stress by hampering readiness to reconcile. Accordingly, religious functioning plays a central role for reconciliation, centrality and mental stress in Rwanda. Implications of this finding concern religious as well as secular professionals. On the one hand religious practitioners should be sensitized on the indirect effect of religious functioning on mental stress and avoid destabilizing believers by proclaiming the image of a strict and punishing God. They should further use their power to foster the readiness to reconcile as many Rwandans might consider religious figures to be legitimate to talk about reconciliation (Assefa, as cited in [36]), whose core concepts (e.g. mercy) are drawn from religious traditions [20]. On the other hand secular professionals should be encouraged to unfurl religious topics and to address religious functioning and associated topics like readiness to reconcile or centrality in therapy and counseling, as data provide evidence, that these issues are important influence factors for mental stress in Rwanda.

### Limitations

Limitations of this study include, that data were self-reported and cross-sectional, therefore not allowing determination of causality. Measures were developed in Western cultures and the study sample was drawn from Kigali, though only about 11% of Rwanda’s population resides in the country’s capital [37]. However, we included respondents from rural and urban neighbourhoods of Kigali in our study. Though data collection took place in an explicit research context and all participants were informed about anonymity, socially desirable response behavior can never be fully prevented. Hence, we controlled for acquiescence by integrating a lie scale into our questionnaire.

## Conclusion

The outcomes of our study show, that rates of PTSD, syndromal depression and syndromal anxiety are elevated in Rwanda. They further reveal that female sex, persecution during the genocide, readiness to reconcile, centrality and religious functioning are important factors for mental stress in the east-central African country. Practical implications of our results are that mental health services in Rwanda should be strengthened to meet the needs of the population and particularly enhance their offers and their availability to women and persons, who were persecuted during the genocide. Data further imply, that the concerted consideration of religious institutions and actors may be beneficial in the development of mental health services, as well as that religious functioning, centrality and readiness to reconcile should be an issue in secular and religious therapy and counseling.
